# 心脏手术后再次心源性休克行心脏移植1例

**DOI:** 10.3760/cma.j.cn121090-20241130-00506

**Published:** 2024-12

**Authors:** 婷 杨, 红雨 叶, 宏开 梁

**Affiliations:** 1 中山市人民医院重症医学科，中山 528400 Department of Critical Care Medicine, Zhongshan City People's Hospital, Zhongshan 528400, China; 2 中山市人民医院心胸外科，中山 528400 Department of Cardiothoracic Surgery, Zhongshan City People's Hospital, Zhongshan 528400, China

## Abstract

因冠心病、二尖瓣返流、三尖瓣返流行“冠状动脉旁路移植、二尖瓣机械瓣置换、三尖瓣成形术”2个月余的患者，再次出现心功能衰竭、心源性休克，主动脉球囊反博术支持1个月后心功能无法恢复，转为体外膜肺氧合（VA-ECMO）支持。VA-ECMO辅助过程中，患者出现复杂的凝血功能障碍，出血和血栓并存，予抗感染、调整抗凝药物及抗感染药物用量、输注血液制品处理，凝血功能好转。心脏移植术后先后发生右心衰竭，多浆膜腔积液（胸腔、腹腔），肺部、血流反复感染，肝功能障碍，肾功能不全，凝血功能障碍，气胸等，经积极治疗后痊愈出院。受者出院后生活质量好，移植心功能基本正常。

## Abstract (en)

Heart failure and cardiogenic shock occurred again on the patient who undergone coronary artery bypass grafting, mechanical mitral valve replacement, and tricuspid valve repair surgery for coronary heart disease, mitral regurgitation, and tricuspid valve regurgitation 2 months ago. His heart function could not recover after Intra-Aortic Balloon Pump（IABP）supported for 1 month, and then Veno-Arterial Extracorporeal Membrane Oxygenation（VA-ECMO）was applied. During the VA-ECMO assisted procedure, complex coagulation dysfunction was present, bleeding and thrombosis was coexisting. Treatment with anti-infective drugs, adjustment dosage of anticoagulant and anti-infective drugs, and infusion of blood products, coagulation function was improved. Cardiac transplantation was applied after VA-ECMO support for 10 days. Right heart failure, multiple serosal fluid accumulation（in the chest and abdominal cavities）, repeated infections in the lungs and blood flow, liver dysfunction, renal dysfunction, coagulation dysfunction, pneumothorax were occurred successively after cardiac transplantation. The patient recovered and discharged from the hospital after active treatment. The recipient's quality of life is good and the transplanted heart function is basically normal.

自1977年Copeland等[1]施行了首例再次心脏移植以来，心脏移植被认为是治疗终末期心脏功能衰竭的惟一有效方法。在国内，我院开展心脏移植手术较早，2000年就开始实施心脏移植手术，积累了一些经验。据回顾，我院心脏移植手术的30 d平均死亡率为9.9％。心脏术后再次出现心功能衰竭、心源性休克同时合并各种并发症需再接受心脏移植的患者数量极少，近5年仅2例。因此，在这种前提下心脏移植手术是否可行且能否成功，是个值得探讨的问题。

## 病例资料

患者男性，57岁。2022年2月因冠心病、二尖瓣返流、三尖瓣返流行“冠状动脉旁路移植、二尖瓣机械瓣置换、三尖瓣成形术”。2022年4月，因心力衰竭、心源性休克入住当地医院，心脏超声检查提示左室收缩功能重度减低，射血分数为20％，三尖瓣大量返流，轻度肺动脉高压。予主动脉球囊反博术（IABP）、机械通气、连续肾脏替代疗法（CRRT）以及药物等对症治疗，期间，多次痰培养找到耐碳青酶烯类鲍曼不动杆菌（CRAB）。2022年5月15日，患者心源性休克无法纠正，转为体外膜肺氧合（VA-ECMO）支持后转入我院，拟行同种异体原位心脏移植手术。

转入我院后，患者镇痛镇静状态，可唤醒，停留气管插管及ECMO动静脉置管。生命体征：体温36.5 °C，心率101次/min，血压116/79 mmHg（去甲肾上腺素0.2 µg·kg^−1^·min^−1^维持）。血常规：白细胞计数（WBC）8.92×10^9^/L，中性粒细胞百分数（NEU％）91.1％，血红蛋白（HGB）88 g/L，血小板计数（PLT）189×10^9^/L。降钙素原4.54 ng/ml；凝血功能：凝血酶原时间（PT）27.3 s，部分活化凝血酶时间（APTT）59.2 s，纤维蛋白原（FIB）4.88 g/L，D-二聚体7.2 mg/L。肺部CT：双肺感染性病变（考虑合并肺水肿）；左下肺支气管异常密度影填充，暂考虑黏液栓；双下肺背侧炎性病变（坠积性肺炎）。完善病原学检查，痰培养及血培养均检出CRAB。此外，同时送检的血液高通量测序（NGS）检出斑疹伤寒立克次体、细环病毒和人类疱疹病毒4型；肺泡灌洗液NGS检出斑疹伤寒立克次体、异栖克雷伯菌和热带念珠菌。经院内讨论，决定先予VA-ECMO维持循环，积极抗感染处理，等待供心和手术时机。抗感染方案为多黏菌素B+磷霉素+多西环素+米卡芬净+阿昔洛韦。在此期间，多次复查心脏超声，均提示左室壁活动普遍减弱，射血分数在10％～20％间波动。

VA-ECMO辅助期间，全程普通肝素抗凝，凝血管理的目标设定为活化凝血时间（ACT）180～200 s或活化部分凝血活酶时间（APTT）为上限值的1.5～2倍。心脏移植前，VA-ECMO辅助10 d，ACT和APTT基本可达标，但临床观察到ECMO置管处及深静脉穿刺点出血、消化道出血，复查血常规，HGB缓慢下降，PLT下降至29×10^9^/L；复查凝血功能，D-二聚体逐渐升高，最高至107 mg/L，纤维蛋白原降解产物最高至335 mg/L，以上临床观察及检验指标提示出血和血栓风险并存。继续予普通肝素抗凝，下调普通肝素用量，同时予以成分输血纠正凝血紊乱，ACT的目标值调整为160～180 s，APTT维持在正常范围。心脏移植术前共输注红细胞3 900 ml，血浆5 300 ml，血小板7个治疗量，冷沉淀10 U，人纤维蛋白原24 g，凝血酶原复合物400 IU ×30瓶。处理后，消化道和穿刺点出血好转，心脏移植术前再次复查相关指标：HGB 79 g/L，PLT 64×10^9^/L，PT 27.3 s，APTT 44.4 s，FIB 1.94 g/L，D-二聚体57.87 mg/L。

2022年5月24日，VA-ECMO辅助时间已经10 d，患者心脏功能仍无好转迹象，近期无发热，复查血常规：WBC 5.94×10^9^/L，NEU％ 66.7％；降钙素原1.19 ng/ml，斑疹伤寒立克次体治疗疗程已足，多次培养血液中未见病原体，凝血功能基本控制在可进行手术的范围内，且有配型成功的供心，决定同种异体原位心脏移植手术。术中见胸骨后心包广泛黏连，组织间隙不清，心脏收缩功能差，全心增大，心肌水肿明显，主动脉根部右侧可见一个大隐静脉吻合口，与周围粘连严重。手术难度大，过程顺利，主动脉阻断113 min，体外循环时间398 min，心脏自动复跳，术中尿量少，停机后右心负荷重，血压偏低，遂继续VA-ECMO辅助。病理结果：镜检见部分心肌细胞稍增大，细胞核增大，可见较多畸形核，核形不规则，染色质呈粗颗粒状；部分肌纤维变性溶解，部分萎缩，心肌间质可见充血，局部可见钙化及多核巨细胞反应；瓣膜水肿、纤维化、玻璃样变，可见异物及多核巨细胞反应；心脏表面血管管壁钙化显著。

术后患者神清，术后第3天，患者循环较前好转，复查心脏超声提示移植心收缩功能正常，射血分数52％，撤离VA-ECMO辅助循环。2022年5月31日，行气管造口术。术后先后并发右心衰竭，多浆膜腔积液（胸腔、腹腔），肺部、血流反复感染，肝功能障碍，肾功能不全，凝血功能障碍，气胸等，根据具体情况，反复调整抗排斥药物、抗感染方案，经床旁CRRT、胸腹腔穿刺引流、输注血制品改善凝血功能、护肝、营养治疗、康复治疗等措施，历经65 d，患者从外科监护室转出，在普通病房继续治疗；2022年9月27日，手术后4个月余，患者出院。出院超声心动图显示：心脏移植术后心包积液（极少量），左心室收缩功能正常。患者出院后能从事轻体力劳动，生活状态好，无乏力、食欲减退，无心悸、胸闷、气促、下肢水肿等症，心功能Ⅰ级。返院复查血液、生化各项指标正常。移植心收缩功能正常，射血分数62％，各瓣膜无反流，舒张功能欠佳。

## 讨论及文献复习

心脏手术后再次出现心功能衰竭，对于患者和医务人员而言都是严峻的考验。一方面，患者的心脏已经历过一次手术，再想通过手术的方式改善心功能的可能性不大；另一方面，这类患者心脏移植手术复杂性极高，前次手术留下的瘢痕组织、胸腔粘连等问题使手术操作更加复杂、风险更高。

ECMO桥接心脏移植是另外一个难题[2]，与非ECMO桥接心脏移植手术患者相比，术前桥接ECMO的患者，无论是短期还是长期预后都更差。其原因可能如下：①需ECMO桥接的患者基础情况往往更差，合并多脏器功能不全的情况更多见；②感染风险增加；③急性失代偿性心源性休克患者使用高剂量血管活性药物；④ECMO运行期间出血和血栓并存。

该例患者病情复杂，因冠心病、二尖瓣返流、三尖瓣返流行“冠状动脉旁路移植、二尖瓣机械瓣置换、三尖瓣成形术”仅仅2个月余再次出现心源性休克，药物已无法维持血流动力学稳定，在IABP的支持下，历经1个月余，心功能无法恢复，还合并了严重的院内感染，转为VA-ECMO进行血流动力学支持。在这样的基础下，进行心脏移植手术风险巨大。

该患者的凝血管理是个难题，在心脏移植前，从临床观察看，患者出现了消化道出血和各穿刺口的出血，凝血监测的指标提示血红蛋白下降，血小板严重下降，APTT，D-二聚体逐渐升高，上述结果提示该患者存在出血和血栓形成并存的矛盾，出血凝血紊乱的原因分析如下：

1. ECMO本身的因素：ECMO支持过程中，最容易出现的并发症中凝血系统的并发症所占的地位尤为重要[3]，由于ECMO期间血液有形成分被破坏，血小板数目减少，在机械因素方面就有了导致凝血功能障碍前提，再加之感染、炎症的影响，也会消耗凝血因子，一方面患者表现为出血倾向，另一方面，又表现为微血栓形成。

2. 肝素相关的凝血功能障碍：肝素是ECMO抗凝中最常选用的抗凝剂，最常见的并发症是出血。该患者出现的胃肠道、穿刺口的出血考虑与肝素的使用相关。患者原本已经存在凝血功能障碍和血小板减少的基础，再使用肝素抗凝出血的风险就大大增加。肝素相关的非致命性出血的最佳治疗是终止肝素使用，但对于必须抗凝的患者而言，特别是在其他药物选择有限的情况下，也可采用减少肝素用量、调整凝血管理的目标、加强胃黏膜保护、局部压迫等手段处理出血问题。

肝素诱导的血小板减少症（heparin-induced thrombocytopenia，HIT）是应用肝素的另外一个并发症，其机制较为复杂，可能与免疫因素有关，也可能与肝素直接激活血小板有关，或者还可能因为血小板抗体的产生导致凝血因子释放，凝血酶生成等[4]。HIT的诊断较为困难，治疗方面首先建议停用肝素，在无法停用肝素的情况下，静脉注射人免疫球蛋白或激素的冲击治疗也是有效的。该例患者我们无法明确血小板严重减少是否有肝素因素，亦无法停用肝素，故选择了静脉注射人免疫球蛋白冲击的治疗方案，效果明显。

3. 感染因素：凝血功能紊乱是严重感染的中心环节，严重感染时，炎症因子的大量产生可过度激活凝血因子[5]–[6]，导致宿主促凝机制上调，抗凝机制下调，纤溶系统抑制，血管内微血栓形成，最终导致弥散性血管内凝血（DIC）、多脏器衰竭的发生[7]。该患者在当地医院时，痰培养多次找到CRAB，提示已经存在院感。入我院时，患者体温正常，考虑与CRRT的降温有关。中性细胞比率高达91.1％，降钙素原4.54 ng/ml，痰培养及血培养均检出CRAB，血液NGS检出斑疹伤寒立克次体、细环病毒和人类疱疹病毒4型，肺泡灌洗液NGS检出斑疹伤寒立克次体、异栖克雷伯菌和热带念珠菌。这些病原体中，最可能致病的是CRAB和斑疹伤寒立克次体，血标本和痰标本里同时检出相同的病原体，可靠性较高。CRAB作为院感常见的细菌，在入住ICU 1个月余，全身多处置管的患者体内发现非常常见，但斑疹伤寒立克次体的检出较为少见，多次询问家属，均无法提供相关流行病学史病史，但既然血液和痰液标本中都检出，说明这个病原体存在及致病的可能性是极大的，需要第一时间覆盖，至于其他如血液中的细环病毒和人类疱疹病毒4型，并没有很强的致病力，但对于重症患者，特别是将来准备行大器官移植的患者，这些病毒也可能发展为致病病原体，因此，也在第一时间予以覆盖。至于真菌方面，肺泡灌洗液NGS检出热带念珠菌，在与外界相同的腔道，念珠菌检出的比率是非常高的，往往不致病，但对于重症患者，因侵袭性真菌感染风险高，国内外指南均推荐可预防性使用棘白菌素抗真菌药物。在这些病原体的影响下，尤其血液中存在病原体的情况下，凝血功能受累的可能性极大，因此，我们认为，患者血小板严重下降，D-二聚体逐渐升高，跟感染的关系密不可分，随着抗感染治疗的深入，凝血指标开始好转，这也从另外一方面说明，感染的控制对凝血的管理至关重要。

4. 药物相关：药物也是影响凝血功能不可回避的因素，如该患者使用的阿昔洛韦，有全血细胞减少症、粒细胞缺乏症、DIC、血小板减少性紫癜等严重不良反应的报道。虽然发生比例不高，但对于原本存在脏器功能不全、尤其是肾功能不全的患者而言，这些不良反应还是应当予以重视。在该患者的处理过程中，药物的问题或许并不是导致凝血障碍的最主要因素，但为了减轻药物的影响，我们还是对阿昔洛韦进行了减量。

对于这类复杂的患者，凝血管理缺乏标准化的方案，在把控血栓形成与出血并发症之间，需要严密的观察和细致的管理，制定个体化的管理方案。

总之，心脏术后再次出现心功能衰竭、心源性休克同时合并多个并发症，需行心脏移植术患者不多见，管理过程缺乏足够的经验和参考依据。但在专业团队的综合评估和调整下，进行个体化的目标管理，还是可以得到良好的结果。
